# Machine Learning Analysis of Alzheimer’s Disease Single-Cell RNA-Sequencing Data across Cortex and Hippocampus Regions

**DOI:** 10.3390/cimb45110544

**Published:** 2023-10-28

**Authors:** Marios G. Krokidis, Aristidis G. Vrahatis, Konstantinos Lazaros, Konstantina Skolariki, Themis P. Exarchos, Panagiotis Vlamos

**Affiliations:** Bioinformatics and Human Electrophysiology Laboratory, Department of Informatics, Ionian University, 49100 Corfu, Greece; aris.vrahatis@ionio.gr (A.G.V.); konlazaros@gmail.com (K.L.); c19skol@ionio.gr (K.S.); exarchos@ionio.gr (T.P.E.); vlamos@ionio.gr (P.V.)

**Keywords:** feature selection, machine learning, Alzheimer’s disease, brain, ensemble method, big data

## Abstract

Advancements in molecular biology have revolutionized our understanding of complex diseases, with Alzheimer’s disease being a prime example. Single-cell sequencing, currently the most suitable technology, facilitates profoundly detailed disease analysis at the cellular level. Prior research has established that the pathology of Alzheimer’s disease varies across different brain regions and cell types. In parallel, only machine learning has the capacity to address the myriad challenges presented by such studies, where the integration of large-scale data and numerous experiments is required to extract meaningful knowledge. Our methodology utilizes single-cell RNA sequencing data from healthy and Alzheimer’s disease (AD) samples, focused on the cortex and hippocampus regions in mice. We designed three distinct case studies and implemented an ensemble feature selection approach through machine learning, also performing an analysis of distinct age-related datasets to unravel age-specific effects, showing differential gene expression patterns within each condition. Important evidence was reported, such as enrichment in central nervous system development and regulation of oligodendrocyte differentiation between the hippocampus and cortex of 6-month-old AD mice as well as regulation of epinephrine secretion and dendritic spine morphogenesis in 15-month-old AD mice. Our outcomes from all three of our case studies illustrate the capacity of machine learning strategies when applied to single-cell data, revealing critical insights into Alzheimer’s disease.

## 1. Introduction

Alzheimer’s disease (AD) is divided into two categories based on the age of onset. Early-onset familial AD is extremely rare, accounting for only 2% of all cases, and appears between the ages of 30 and 60, with more than half of patients having a genetic predisposition. Conversely, late-onset AD is the most common form of the disease. It also has some genetic predisposition. However, it carries several gene polymorphisms, some of which have not yet been identified, and which, either individually or in combination, increase the likelihood of disease occurrence [1]. There is a compelling need for disease-modifying therapies that may prevent or slow the rate of progression. Important progress has been achieved in recent years to elucidate key aspects of the underlying pathophysiology of AD and new therapeutic strategies are still being actively developed and tested. Recently, aducanumab became the first FDA-approved drug to address the underlying biology of the disease. This monoclonal antibody reduces beta-amyloid plaques, which are reasonably likely to lead to a reduction in clinical decline due to Alzheimer’s disease [2].

RNA sequencing, often referred to as RNA-seq, is a powerful technique used to analyze the transcriptome of a biological sample. The transcriptome represents the entire set of RNA molecules, including mRNA (messenger RNA), non-coding RNA, and other functional RNA molecules present in a specific cell or tissue at a given time. As scRNA-seq platforms become more readily available on the commercial market and bioinformatics methods continue to advance, the field has now reached a stage where any biomedical researcher or clinician can leverage scRNA-seq to make groundbreaking discoveries [3]. Data integration serves as an essential tool for conducting collective analyses on single-cell RNA sequencing (scRNA-seq) data. The principal objective of this process is the synthesis of multiple datasets originating from varied sources into a single, unified, and cohesive dataset [4]. This amalgamation augments the resolution and depth of analysis and it also enables a more precise comprehension of trends and patterns obscured within the data. The inclusion of diverse data sources is critical to research since it fortifies the robustness of the analysis of biological phenomena and propels a stronger understanding of the intrinsic mechanisms linked to the phenomena under investigation [5].

Twenty-two large-scale AD gene expression datasets were utilized to explore a consistent underlying portrait of AD gene expression across multiple brain regions, indicating dysregulation of inositol trisphosphate kinase, astrocyte specific intermediate filament protein, GFAP, and RHOQ [6]. Proteomic analysis of more than 1000 brain tissues was performed to assess differences in AD-related protein co-expression across cohorts and brain regions, with half of the protein co-expression modules being absent in RNA networks from the same cohorts and brain regions. In that study, the *APOE ε4* allele regulated the matrisome module and the rate of cognitive decline influenced the MAPK/metabolism module [7]. Cross-study large-scale transcriptomic analyses of 230 postmortem prefrontal cortex tissues derived from AD patients were carried out along with module co-expression analysis and MCC gene ranking in common pathways, indicating that many of the lost synaptic genes that participate in AD encode protein networks related to cognitive stability and synaptic function, as well as to immune response pathways and glutamatergic and GABAergic transmission [8]. A novel deep neural network-based prediction model for multiple heterogeneous omics datasets was developed, with enhanced performance compared to conventional ML algorithms, while various comparative experiments changing each component of the method were executed to test the proposed method [9]. AD-related genes from blood samples can also contribute to the development of blood-based AD diagnostic and treatment tools. The expression values of AD-related genes obtained from blood samples of ADNI, ANM1, and ANM2 were classified using five feature selection methods and five classifiers; AD-related genes were enriched with several pathways, showing their immune, inflammation, energy metabolism, and Wnt signaling involvement [10]. In a recent study by our group, we also examined the impact of an ensemble pipeline including the SCALEX integration tool in the context of single-cell AD studies from the peripheral blood of both AD patients with an amyloid-positive status and healthy amyloid-negative controls [11].

A noteworthy hurdle encountered during data integration is the occurrence of batch effects, which are inherent in the sequencing process. Batch effects constitute a prevalent concern for scRNA-seq integrative analyses as they instigate systematic variance in sample groups, which is not attributable to biological factors, but rather to technical noise [12]. These effects can result in erroneous conclusions and confound true biological cell heterogeneity. Given the plethora of potential sources from which batch effects can originate, addressing them during data integration is of paramount importance to ensuring the reliability and accuracy of the resultant data.

Numerous machine learning techniques have been developed to integrate multi-omics data. These methods encompass Bayesian approaches, heterogeneous graph approaches like matrix factorization-based algorithms, deep learning approaches, and various other machine learning techniques [13]. The datasets utilized for the purposes of this research were obtained from the scREAD database [14]. This database encompasses both scRNA-seq and snRNA-seq datasets derived from postmortem human brain tissue exhibiting Alzheimer’s Disease (AD) and mouse models with AD pathology. It also includes control datasets sourced from healthy, non-AD samples. The overarching aim of the study was to examine three distinct scenarios. Firstly, the intention was to assess the differences in gene expression between healthy control brain cells and AD-affected cellular environments in the hippocampus. The second objective was to analyze gene expression disparities between healthy control cells and AD-affected ones within the cortex. Lastly, we evaluated transcriptome differences between AD-affected cells located in both regions of the mouse model brain, that is, the cortex and the hippocampus. Furthermore, analysis of age-related datasets was performed to unravel important insights based on the equilibrium in age distribution among healthy and diseased tissues. Gene set enrichment analysis as a computational method determines whether a defined set of genes shows statistically significant, concordant differences between two biological states. However, the differentially expressed genes produced by scRNA-seq methods and analyzed by GSEA often contain biologically irrelevant data lacking discriminatory power to classify the groups present in the dataset as the analysis of such data poses a significant challenge due to its sheer size and complexity. We used feature selection to design three distinct case studies and implemented an ensemble FS approach through machine learning, a powerful process in which a subset of relevant features is selected from a larger dataset using specific algorithms for different tasks, such as classification, clustering, and regression analysis. Thanks to this approach, our pipeline effectively removed batch effects, which ensured the reliability and accuracy of our results. Without this process, the dataset would have contained batch effects, which could have compromised the validity of any analysis framework used. The present study aimed to underscore the efficacy and potential of refined feature selection strategies applied to single-cell data.

## 2. Materials and Methods

### 2.1. scRNA-Seq Data Integration

Our methodology utilized single-cell RNA sequencing data from healthy and Alzheimer’s disease (AD) samples, focused on the cortex and hippocampus regions. We designed three distinct case studies and implemented an ensemble feature selection approach through machine learning. Our strategy aimed to identify the most influential genes from diverse brain regions in the context of AD.

A total of 14 datasets sourced from the scREAD database [14] were employed in the analysis, comprising cells from the hippocampus of mouse models, a total of 18,269 cells. Within these datasets, 4 represented healthy control cells, while the remaining 10 were indicative of cells presenting with AD pathology. Furthermore, 8 datasets comprising cells from the cortex of the mouse models were utilized, totaling 92,859 cells. Among these, 2 datasets corresponded to healthy controls, while the remaining 6 represented cells with AD pathology.

For all three cases, we merged and preprocessed the related datasets using Seurat’s [15] standard pipeline and subsequently performed data integration/batch effect correction using Seurat’s CCA method. This technique anticipates correspondences or mutually occurring biological states within a subset of single cells across different groups. The process is streamlined into four primary steps. Initially, a CCA is conducted. CCA identifies shared variation sources between different conditions or groups, functioning similarly to principal component analysis (PCA). This technique targets significant sources of data variation that are shared or conserved across different conditions or groups, employing the 2000 most variable genes across all datasets/batches. This initial step aids in the rough alignment of cells based on the most significant shared variation sources. Subsequently, the process moves to identify “anchors” or mutual nearest neighbors (MNNs) across datasets, acknowledging the potential for erroneous anchor identification. Anchors are determined through an analysis of the cell’s closest neighbor in alternate conditions, with in this analysis were founded on gene expression values. A reverse analysis is subsequently conducted, and if two cells emerge as mutual nearest neighbors, they are marked as anchors to tether the two datasets together. Differences in expression values between cells within an MNN pair provide an estimation of the batch effect, refined by averaging across multiple pairs. A correction vector is then derived and applied to the expression values to execute batch correction. Following this, an anchor filtering process is initiated to discard incorrect anchors. Similarities between anchor pairs are assessed through the overlap in their local neighborhoods, with incorrect anchors yielding low scores. The adjacency of the cells’ nearest neighbors is a key factor in this step. Finally, the conditions or datasets are integrated. The anchors and corresponding scores are utilized to transform the cell expression values, facilitating the integration of the conditions/datasets. In situations where specific cell types are present in one dataset but absent in another, these cells will still manifest as a distinct sample-specific cluster.

It warrants noting that, for the first scenario, the integration process encompassed the 14 datasets derived from the hippocampus. For the second scenario, the eight datasets derived from the cortex were integrated. Finally, for the third scenario, a total of 16 datasets, comprising AD-affected cells from both the cortex and the hippocampus of the mouse model brain, were integrated. Consequently, following the integration process, three distinct datasets were obtained. The first integrated dataset, encompassing both AD and control cells from the hippocampus region, yielded a total of 12,650 cells post preprocessing and integration. The second integrated dataset, comprising both AD and control cells from the cortex region, resulted in a total of 75,969 cells following the preprocessing and integration process. The final integrated dataset, which included AD cells from both the cortex and hippocampus regions of the mouse model brains, amassed a total of 54,659 cells. Significantly, each integrated dataset incorporated the 2000 genes demonstrating the highest degree of variability, in line with Seurat’s processing pipeline, underscoring the meticulous methodological approach applied in this analysis.

### 2.2. Feature Selection Procedure

Based on prior research, it was established that amidst the thousands of genes present in scRNA-seq datasets, only a select few hundred truly dominate the biological phenomena under investigation [16,17]. Recognizing this, a hybrid feature selection procedure was deployed on all three datasets obtained post integration. Initially, for each case, the 2000 genes were arranged in accordance with their differential expression gene (DEG) score for the two conditions being examined in each of the three scenarios. This ranking was achieved through a Wilcoxon rank-sum test [18], a non-parametric test examining if values in one group of interest are significantly greater or smaller than those in another group of interest. In the first two scenarios, these groups were represented by control and AD samples, while in the third scenario, they corresponded to the two previously specified brain regions, the cortex and the hippocampus.

**Figure 1:**
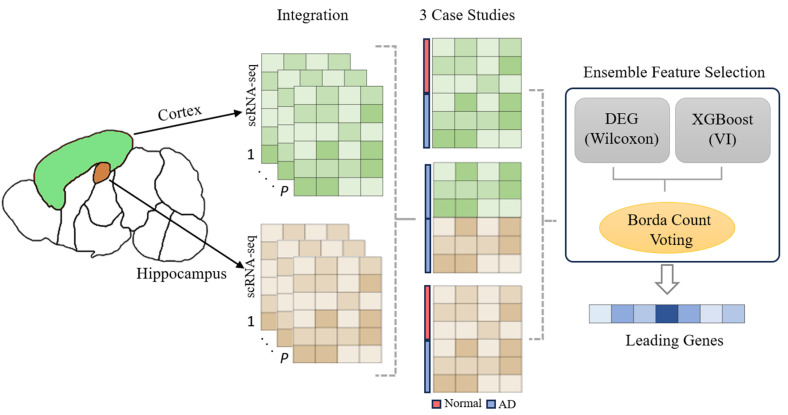
Data integration and feature selection pipeline. Data integration was performed using Seurat’s CCA method. For the three case studies created through integration, Wilcoxon’s rank-sum test was used for DEG identification and XgBoost’s variable importance criterion was used to identify important genes for distinguishing between the 2 conditions of each case. The top 100 genes from each case were kept after obtaining a Borda consensus list of the DEGs and XGBoost important genes for each case study.

Subsequently, the 2000 genes were ranked by importance employing an xgboost classifier [19]. In all three cases, “gain” importance was utilized to order genes from most to least significant, while all other parameters were maintained at their default values. Gain measures the relative contribution of a particular feature to the model, computed by considering each feature’s contribution for each tree in the model. A feature’s higher metric value in comparison to another feature implies its heightened importance for generating a precise prediction pertinent to the condition of interest. For each case, these two ranked lists, acquired from the Wilcoxon rank-sum test and xgboost’s feature importance criterion, are then combined amalgamated into a consensus list through Borda’s rank aggregation method [20]. This method, named after the French politician Jean-Charles de Borda, relies on the ranking of elements by multiple classifiers, founded on a specific criterion such as the probability of an output value being accurate or the importance of a feature. Each element is assigned a rank value between 0 and m − 1, with m representing the total number of elements. The ranks assigned by each classifier are subsequently totaled to calculate a cumulative grade or rank for each element. Elements are then arranged in descending order based on their cumulative grade, with random selection employed in the case of a tie. The fundamental principle behind the aggregation method is to bolster the robustness of the feature selection procedure while minimizing bias that might arise from the sole use of a single feature selection method. Ultimately, for each case, the top 100 genes from the consensus list were selected for further analysis, ensuring the most significant genes were the focus of the subsequent exploration. The pipeline we followed in order to perform data integration/batch effect correction as well as feature selection is summarized in Figure 1. In summary:

- We procured 14 datasets encompassing cells from the hippocampus region of the mouse brain and an additional 8 datasets focusing on cells from the mouse cortex region.
- These datasets were organized into three distinct case studies; firstly, an analysis comparing healthy control cells with AD cells derived from the mouse cortex; secondly, a similar evaluation focused on the mouse hippocampus; and thirdly, a case study differentiating AD cells from both the mouse hippocampus and cortex brain regions.
- For all three case studies, the datasets utilized were integrated through the use of Seurat’s CCA method (2000 HVGs were also kept in this step).
- Feature ranking took place for each of the case studies after integration, using both the Wilcoxon rank-sum test as well as XgBoost’s variable importance (VI) criterion in order to rank features from most to least important. Xgboost was utilized with each case’s label of interest (control vs. disease and cortex vs. hippocampus) in order to rank genes through the use of gain VI. The same applies for the Wilcoxon rank-sum test.
- These two lists obtained through the use of the algorithms mentioned above were subsequently combined into a single consensus list through the use of the Borda rank-based count voting scheme. From this consensus list, the top 100 genes were kept for the subsequent steps of our analysis.

## 3. Results and Discussion

**Figure 2:**
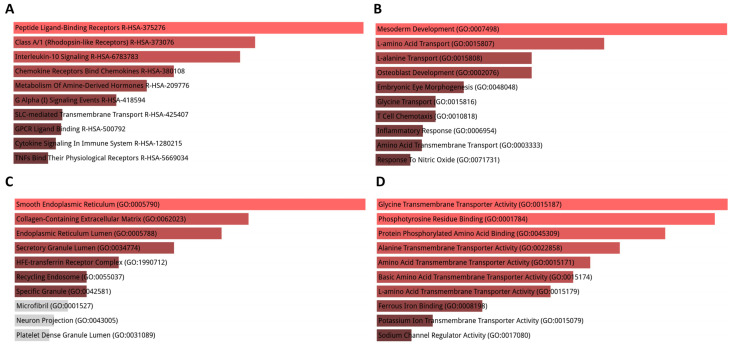
GO term and pathway enrichment analysis performed using Enrichr on cortex DEGs. (**A**) Top 10 enriched Reactome pathways for DEGs. (**B**) Top 10 enriched biological processes for DEGs. (**C**) Top 10 enriched cellular components for DEGs. (**D**) Top 10 enriched molecular functions for DEGs. Further information is provided in Tables S1–S4.

We conducted an enrichment analysis using Enrichr [21,22] to explore the high-level functions and utilities of the biological system according to the exported genes. GO function and Reactome [23] pathway enrichment analyses were performed for the most of the provided cortex DEGs (Tables S1–S4). Reactome pathway analysis showed that DEGs were mainly enriched in pathways such as transmembrane transport, synthesis of GDP-mannose, extrinsic pathway of fibrin clot formation, and metabolism of amine-derived hormones and insulin-like growth factor-2 mRNA binding proteins (Figure 2A). The enriched GO terms were divided into biological process (BP), molecular function (MF), and cellular component (CC) ontologies. The results of GO analysis revealed that DEGs were mainly enriched in BPs including L-alanine transport, glycine transport, T cell chemotaxis, and thiamine transmembrane transport (Figure 2B). CC analysis indicated that DEGs were significantly enriched in the smooth endoplasmic reticulum, collagen-containing extracellular matrix, and protein kinase complex (Figure 2C). As for MF, DEGs were enriched in glycine and alanine transmembrane transporter activity, oxoreductase activity, ATP-activated potassium channel activity, and acetylcholine receptor inhibitor activity (Figure 2D).

More precisely, based on our analysis, *SLC38A5* encodes a protein which serves as a transporter of glutamine, asparagine, histidine, serine, alanine, and glycine across the cell membrane. The role of solute carrier transporters in neurodegenerative disorders such as Alzheimer’s disease, amyotrophic lateral sclerosis, Huntington’s disease, and Parkinson’s diseases is highly reported [24]. In neurons from the SLC family, neurotransmitters are considered to be the cause of neurodegenerative disorders such as schizophrenia, epilepsy, depression, and Parkinson’s disease (DAT), as well as amyotrophic lateral sclerosis (GLT) [25].

**Figure 3:**
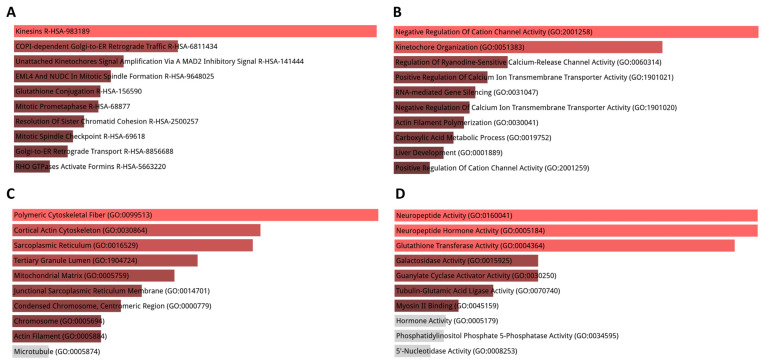
GO term and pathway enrichment analysis performed using Enrichr on DEGs in the hippocampus. (**A**) Top 10 enriched Reactome pathways for DEGs. (**B**) Top 10 enriched biological processes for DEGs. (**C**) Top 10 enriched cellular components for DEGs. (**D**) Top 10 enriched molecular functions for DEGs. Further information is provided in Tables S5–S8.

In the hippocampus, Reactome pathway analysis showed that DEGs were mainly enriched in pathways such as synthesis of lipoxins, choline catabolism, transcription of neuronal ligands, kinesins, synthesis of 5-eicosatetraenoic, and acetylcholine-related regulation of insulin secretion (Figure 3A). The results of GO analysis revealed that DEGs were mainly enriched in BPs including kinetochore organization, negative regulation of cation channel activity, regulation of epinephrine secretion, L-ascorbic acid metabolism processes, and regulation of guanylate cyclase activity (Figure 3B). CC analysis indicated that DEGs were significantly enriched in the junctional sarcoplasmic reticulum membrane, axonal growth cone, cortical actin cytoskeleton tertiary granule lumen, and AMPA glutamate receptor complex (Figure 3C). As for MF, DEGs were enriched in neuropeptide activity, guanylate cyclase activator and tubulin-glutamic acid ligase activity, neuropeptide hormone and glutathione transferase activity, as well as myosin II binding and phosphatidylinositol phosphate 5-phosphatase activity (Figure 3D).

*SLC38A5* encodes a protein which serves as a transporter of glutamine, asparagine, histidine, serine, alanine, and glycine across the cell membrane. Glutamine is an essential neurotransmitter precursor and is involved in various metabolic pathways, including the synthesis of neurotransmitters like glutamate and GABA. The precise role of SLC38A5 in the brain is not fully understood, but it is believed to be crucial for supporting neurotransmission, synaptic function, and overall brain metabolism. Dysregulation or mutations in SLC38A5, as in the rest of the SLC family, may have implications for brain health and could be associated with certain neurological disorders, although research in this area is still ongoing [26]. The protein encoded by *HPDL* localizes to the mitochondria, where it may function as 4-hydroxyphenylpyruvate dioxygenase. Numerous studies have associated the HPDL gene with a broad range of neurodegenerative phenotypes across various clinical scenarios. The reported cases include individuals who exhibited symptoms spanning from neonatal encephalopathy to adolescent-onset uncomplicated spastic paraplegia [27].

The LIPT1 gene encodes for lipoyltransferase 1, which is an enzyme involved in the biosynthesis of lipoic acid. Mutations in the lipoyltransferase LIPT1 gene cause a fatal disease associated with a specific lipoylation defect of the 2-ketoacid dehydrogenase complexes [28]. Lipoic acid is an essential co-factor for various enzyme complexes involved in cellular energy metabolism, particularly in the citric acid cycle and the alpha-ketoglutarate dehydrogenase complex. It also plays a crucial role in generating ATP, which is the primary energy source for cells, including neurons. FASTKD5 encodes for a protein called Fas-activated serine-threonine kinase domain-containing protein 5, a member of the FAST kinase domain-containing (FASTKD) family, which is involved in various cellular processes, particularly in the regulation of mitochondrial-related pathways and apoptosis and may impact neuronal survival and homeostasis [29]. Furthermore, NLRX1 translocates to the mitochondrial matrix and associates with mitochondrial FASTKD5 [30]; it potentially holds significance in neurodegenerative disorders, particularly those characterized by necrosis as a prominent factor. *NLRX1* is involved in regulating mitochondrial dynamics and quality control and promotes mitochondrial fission. The encoded protein is involved in modulating inflammatory responses in neurons while the dysregulation of neuroinflammatory processes is implicated in various neurodegenerative diseases and could contribute to neuronal survival in response to stress [31].

**Figure 4:**
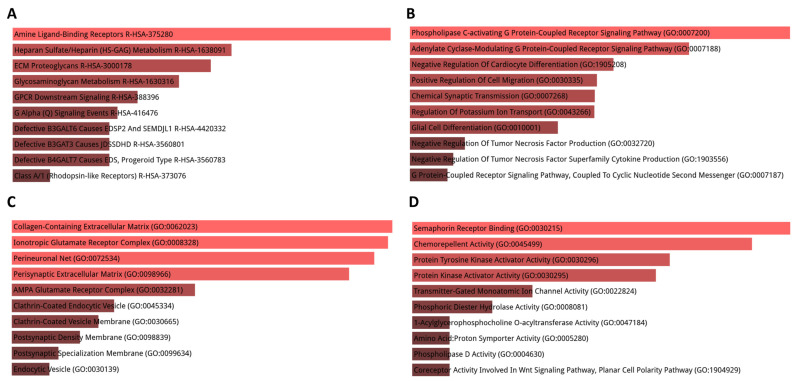
GO term and pathway enrichment analysis performed using Enrichr on DEGs in the cortex compared to the hippocampus. (**A**) Top 10 enriched Reactome pathways for DEGs. (**B**) Top 10 enriched biological processes for DEGs. (**C**) Top 10 enriched cellular components for DEGs. (**D**) Top 10 enriched molecular functions for DEGs. Further information is provided in Tables S9–S12.

Comparing the cortex with the hippocampus, Reactome pathway analysis indicated that DEGs were mainly enriched in pathways such as tachykinin receptor binding, muscarinic acetylcholine receptors, dopamine receptors, synthesis of lipoxins, and activation of TRKA receptors, a family of tyrosine kinases that regulates synaptic strength and plasticity in the mammalian nervous system ((Figure 4A). The results of GO analysis demonstrated that DEGs were mainly enriched in BPs including the adenylate cyclase-activating adrenergic receptor signaling pathway, positive regulation of gap junction assembly, regulation of epinephrine secretion, loading of neurotransmitters into synaptic vesicles, and regulation of nitric oxide-mediated signal transduction (Figure 4B). CC analysis indicated that DEGs were significantly enriched in the perineuronal net, perisynaptic extracellular matrix, and multivesicular dense-core vesicle, as well as ionotropic and AMPA glutamate receptor complexes and clathrin-sculpted gamma-aminobutyric acid transport vesicle (Figure 4C). As for MF, DEGs were enriched in 1-acylglycerophosphocholine o-acyltransferase activity, phospholipase D activity, G protein-coupled acetylcholine receptor activity, and xylosyltransferase activity (Figure 4D).

**Figure 5:**
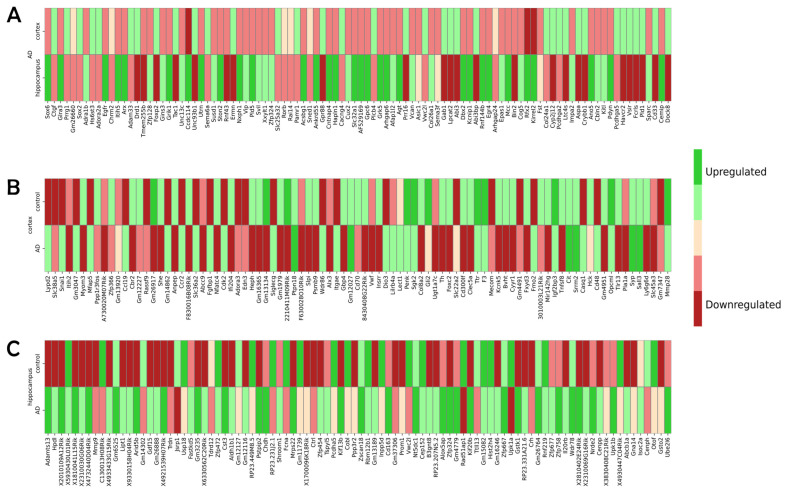
Heatmaps of up- and downregulated genes across the three case studies. (**A**) Up- and downregulated genes for AD cells in both the cortex and hippocampus regions of the mouse brain. (**B**) Down- and upregulated genes for AD and control cells within the cortex region of the mouse brain. (**C**) Up- and downregulated genes for AD and control cells in the mouse hippocampus region. Across all depictions, a deeper green color indicates pronounced upregulation, while a deep red signifies marked downregulation. Milder upregulation is represented by a pale green, whereas moderate downregulation is portrayed by a light red. Genes maintaining consistent expression levels are delineated in a soft yellow.

We also generated three distinct heatmaps corresponding to each of the case studies conducted. These heatmaps serve to elucidate the differential gene expression patterns between the two conditions under investigation in each scenario. In the provided graphical illustrations, genes manifesting pronounced upregulation are designated with a deeper green color. Those with moderate upregulation are indicated by a lighter shade of green. Conversely, genes evidencing moderate downregulation are characterized by pale red, while those with pronounced downregulation bear a deeper red coloration. Genes that remain unaltered in their regulation are rendered in a light yellow, as shown in Figure 5.

The *SOX6* gene is a member of the SOX family of transcription factors, which are crucial for regulating gene expression during various stages of development and cell differentiation. It plays a vital role in multiple biological processes, particularly in skeletal and cardiac muscle development, chondrogenesis (formation of cartilage), and the development of the central nervous system [32]. The encoded protein plays a crucial role as a transcriptional activator, contributing to the normal development of the central nervous system, chondrogenesis, and the maintenance of cardiac and skeletal muscle cells. In pallial and subpallial progenitors, SOX6 and SOX5 exhibited predominantly mutually exclusive expression. Subsequently, in postmitotic neuronal progeny, they maintained this mutually exclusive pattern but in reverse. When SOX6 was lost from pallial progenitors, it led to the improper expression of developmental controls that are typically restricted to the subpallium. As a consequence, these progenitors acquired a mixed dorsal–ventral identity [33]. Numerous transcription factors, including Nkx2-1, Lhx6, and Sox6, have been identified as essential for the differentiation of progenitor cells into cortical parvalbumin-expressing (Pvalb+) neurons [34]. The expression of Sox6 helps differentiate dopamine neurons in the substantia nigra into two distinct groups: dorsally and ventrally biased neurons. These two groups exhibit unique properties and embryonic origins [35].

*CTGF*, known as connective tissue growth factor, plays a crucial role in tissue development and repair through various cellular activities. It promotes the synthesis of essential extracellular matrix components like collagen and fibronectin, which are vital for maintaining tissue structure and integrity. Additionally, it regulates cell migration, adhesion, and proliferation, further contributing to its significance in tissue-related processes. Previously reported studies have indicated elevated levels of CTGF/CCN2 in the spinal cord of ALS patients. However, there is currently no evidence regarding the role of CTGF/CCN2 in neurodegenerative diseases like ALS, where skeletal muscle alterations appear to be a consequence of early pathological denervation [36]. In AD patients, CTGF/CCN2 expression is increased in astrocytes surrounding plaques. However, the precise role of CTGF in AD pathogenesis is not yet fully understood. Recent findings suggest that CTGF facilitates the uptake and subsequent degradation of Aβ (amyloid-beta) within primary glia and neuroblastoma cells. It appears that CTGF enhances extracellular Aβ degradation through membrane-bound matrix metalloproteinase-14 (MMP14) in glial cells and extracellular MMP13 in neurons [37].

Semaphorins, initially recognized as guidance cues for developing axons, have a pivotal role in shaping the nervous system during development. They actively participate in various processes, such as neuronal proliferation, migration, neuritogenesis, and synapse formation [38]. Sema6A functions as a gatekeeper, regulating the boundary between the peripheral nervous system (PNS) and the central nervous system (CNS) in both the ventral and dorsal directions. It plays a crucial role in the clustering of boundary cap cells at the PNS/CNS interface, ensuring that motoneurons are retained within the ventral spinal cord and do not stream out [39]. Cut-like homeobox 2 (Cux2) is a transcription factor that plays a role in dendrite and spine development, as well as synapse formation in projection neurons located in the upper neocortical layers of mice. When CUX2 is deficient, it leads to increased facilitation of excitatory synaptic transmission onto the hippocampus and heightened susceptibility to seizures induced by kainite [40]. Evidence of early-determined Cux2-positive neuronal precursors has been observed in the subventricular zone (SVZ) and intermediate zone (IZ), as well as in upper-layer neurons. This discovery suggests that the laminar determination of upper-cortical-layer neurons takes place during the earliest stages of corticogenesis [41]. Furthermore, the expression of Cux2 in cortical layers II–IV can be modulated through the interaction of Cux2-E1 and Lhx2. It is linked to the development of neurodevelopmental disorders like autism and schizophrenia [42]. GRK5, a multifunctional protein expressed in various cell types, is found in single or multiple subcellular compartments. Acting as a bridging factor, GRK5 plays a crucial role in regulating signaling in pathophysiological conditions, ensuring the coordination between cardiovascular and neurophysiological complications associated with aging [43], while an association has been reported with early AD-like pathologic and behavioral changes in GRK5 KO mice [44].

**Figure 6:**
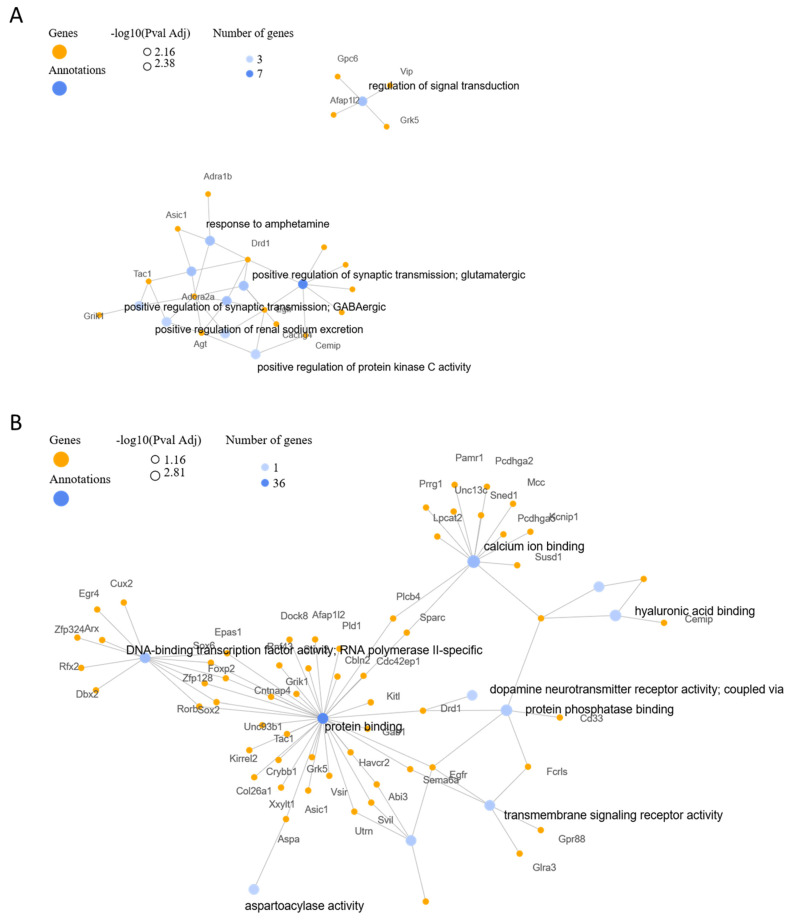
Gene annotation cluster networks. Visualizations generated for 100 top terms of related categories identified in DEG lists and gene annotation cluster networks for (**A**) GO biological processes and (**B**) GO molecular functions.

Further ontological analysis and gene annotation cluster networks were implemented using GeneCodis4, as shown in Figure 6. BP analysis showed an involvement in the regulation of synaptic vehicles exocytosis, cerebral cortex GABAergic interneuron migration, and regulation of norepinephrine secretion. CC analysis indicated that the genes participated in synapses, extracellular regions, glutamatergic synapses, and dendrite and cell surfaces. Lastly, MF analysis revealed an involvement in calcium ion and protein phosphatase binding, DNA-binding transcription factor activity, and actin filament binding.

Brain cells are intricate biological entities with functions determined by conserved gene expression programs at the molecular level. To comprehend the molecular identity of neural cell types, it is necessary to analyze thousands of genes, thereby revealing subtle differentiations between cells [45]. Single-cell sequencing datasets encompass thousands of features, measured across thousands to millions of individual cells. The analysis of such data demands advanced computational procedures, and the chosen analysis pipelines can profoundly influence the outcomes. The availability of open-source software packages enables neurobiologists to employ sophisticated analytic strategies for their research [46]. Transcriptomics in the mouse brain has been instrumental in advancing our understanding of brain biology, neurodevelopmental processes, neural circuitry, and the molecular mechanisms underlying neurological and psychiatric disorders. It enables researchers to identify key genes and pathways involved in brain function and dysfunction, ultimately paving the way for the development of potential therapeutic targets for neurological diseases. The mammalian brain exhibits a high level of complexity, involving numerous cell types with diverse functions. However, our understanding of how each cell type is affected during aging remains largely unclear. To address this, researchers conducted a single-cell transcriptomic analysis of young and old mouse brains, generating comprehensive datasets of aging-related genes, pathways, and ligand–receptor interactions in nearly all brain cell types [47]. The analysis unveiled gene signatures that show coordinated changes across different cell types, as well as gene sets that are specifically regulated in a cell-type dependent manner, sometimes even exhibiting opposite trends. These findings suggest that aging does not trigger a uniform program but rather leads to distinct transcriptional changes in each cell population.

Through the reconstruction of thousands of clones, fate-restricted progenitor cells in the mouse hippocampal neuroepithelium were discovered [48]. The study also revealed that microglia originate from a limited number of primitive myeloid precursors, which undergo massive expansion to generate widely dispersed progeny. Additionally, a combination of spatial transcriptomics with clonal barcoding was carried out to disentangle migration patterns of clonally related cells in densely labeled tissue sections. A single-cell RNA sequencing (scRNA-seq) dataset was used to characterize vascular and vessel-associated cell types and subtypes in the mouse brain and lungs. The dataset included 3436 single cell transcriptomes from the mouse brain, resulting in the identification of 15 distinct clusters representing various cell (sub)types [49]. Additionally, the dataset encompassed 1504 single cell transcriptomes from the mouse lungs, leading to the identification of 17 cell clusters. Altogether, this comprehensive molecular atlas provided valuable insights into the diversity of vascular and vessel-associated cell types in the mouse brain and lungs. Using spatially resolved single-cell transcriptomics, a high-resolution cell atlas of brain aging in the frontal cortex and striatum was presented, tracking changes in gene expression and the spatial organization of major cell types throughout the mouse lifespan [50]. More pronounced alterations in cell state, gene expression, and spatial organization among non-neuronal cells compared to neurons were detected, uncovering distinctive molecular and spatial signatures of glial and immune cell activation during aging. Interestingly, similarities and notable differences in cell activation patterns induced by aging and systemic inflammatory challenges were observed, shedding light on the mechanisms of age-related decline and inflammation in the brain.

**Figure 7:**
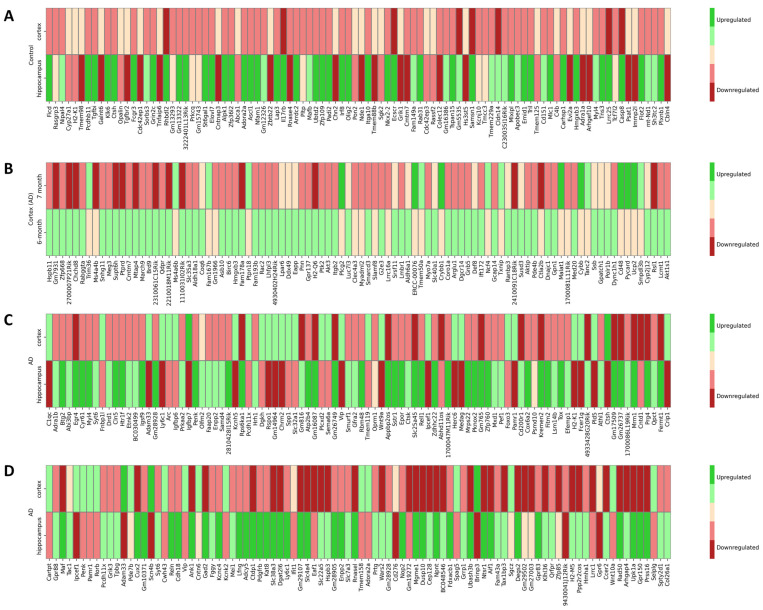
Heatmaps of up- and downregulated genes across the distinct age-related datasets. (**A**) Up- and downregulated genes for control cells in both 6-month-old cortex and 6-month-old hippocampus regions of mouse brain; datasets AD00301 and AD00702. (**B**) Up- and downregulated genes for AD cells in the cortex region of 6-month-old and 7-month-old mouse brain; datasets AD00303 and AD00705. (**C**) Up- and downregulated genes for AD cells in both 15-month-old cortex and 15-month hippocampus regions of mouse brain; datasets AD00307 and AD00714. (**D**) Up- and downregulated genes for AD cells in both 15-month-old cortex and 15-month-old hippocampus regions of mouse brain; datasets AD00308 and AD00715. Across all depictions, a deeper green color indicates pronounced upregulation, while a deep red signifies marked downregulation. Milder upregulation is represented by a pale green, whereas moderate downregulation is portrayed by a light red. Genes maintaining consistent expression levels are delineated in a soft yellow.

In our machine learning pipeline, which integrated multiple single-cell RNA seq datasets from the scREAD database, we utilized samples spanning an age range from 6 to 15 months. At an initial glance, this age disparity might raise concerns, especially given the known influence of age on AD progression. However, a deeper dive into our dataset reveals a crucial balance. Specifically, there is an equilibrium in age distribution among both the control and disease samples, ensuring that age-related biases do not disproportionately influence one group over the other. To overcome potential concerns, we further performed feature selection in specific age groups and analyzed age-specific effects to underscore the potential of machine learning in handling and integrating data from the same age, adding another layer of validity to our approach. Data integration and feature selection in 6-month-old cortex and 6-month-old hippocampus tissue in control mice (datasets AD00301 and AD00702, 5XFAD mice) was executed and the top 100 genes from each case were kept after obtaining a Borda consensus list of DEGs and XGBoost important genes for each case study, as Figure 7A depicts. Six distinct heatmaps corresponding to each of the case studies conducted are presented in Figure 7, showing the differential gene expression patterns between each condition. The five categories in the heatmaps are visually represented with a color spectrum ranging from deep red to deep green; deep red signifies strong downregulation, deep green indicates strong upregulation, and intermediate colors depict degrees of moderate down- or upregulation. Enrichment analysis was conducted to assess the high-level functions and utilities of the biological system according to the exported genes in 6-month-old control datasets. The results of GO analysis revealed that DEGs were mainly enriched in BPs including central nervous system development, positive regulation of pseudopodium assembly, regulation of oligodentrocyte differentiation, and negative regulation of peptidyl-serine phosphorylation (Figure S2). CC analysis indicated that DEGs were significantly enriched in the ionotropic glutamate receptor complex, postsynaptic density membrane, and astrocyte projection (Figure S3), and MF analysis showed an enrichment in ionotropic glutamate receptor activity, low-density lipoprotein particle binding, neurotransmitter receptor activity involved in the regulation of postsynaptic membrane potential, and transmitter-gated monoatomic ion channel activity (Figure S4). Comparison between a 6-month-old AD cortex and a 7-month-old AD cortex (datasets AD00303 and AD00705, 5XFAD mice) was also performed, and the top 100 genes were provided (Figure 7B). BP analysis depicted that DEGs were significantly enriched in the regulation of neutrophil migration, positive regulation of dendritic cell cytokine production, and positive regulation of transcription elongation by RNA polymerase II (Figure S5). CC analysis showed an enrichment in the NADPH oxidase complex, polymeric cytoskeletal fiber, and extracellular membrane-bounded organelle, while MF analysis showed an enrichment in oxidoreductase activity and protein tyrosine phosphatase activity, respectively (Figures S6 and S7).

Moreover, data integration and feature selection in 15-month-old cortex and 15-month-old hippocampus tissue in AD mice (datasets AD00307 and AD00714, Trem2_KO mice) was carried out and the top 100 genes from each case were kept a following similar approach (Borda consensus and XGBoost variable importance), as presented in Figure 7C. GO analysis revealed that DEGs were mainly enriched in BPs including the phospholipase C-activating G protein-coupled receptor signaling pathway, membrane invagination, and adenyate cyclase-inhibiting G protein-coupled acetylcholine receptor signaling pathway (Figure S8). CC analysis highlighted that DEGs were significantly enriched in neuron projection, dendrites, and axons (Figure S9), while MF analysis showed an enrichment in cysteine-type endopeptidase activator, G protein-coupled serotonin activity, voltage-gated monoatomic cation channel activity, and neuropeptide hormone activity (Figure S10). To strengthen our exports, we also performed an additional analysis comparing a further dataset of 15-month-old cortex and 15-month-old hippocampus tissue of diseased mice (AD00308 and AD00715, 5XFAD mice); the top 100 genes are highlighted in Figure 7D. BP analysis showed that DEGs were significantly enriched in the positive regulation of kinase activation, regulation of epinephrine secretion, and regulation of dendritic spine morphogenesis (Figure S11). CC analysis indicated an enrichment in the sodium channel complex and neuron projection, whereas MF analysis indicated an enrichment in G-protein-coupled receptor activity, exonuclease activity, and potassium channel and neuropeptide activity, respectively (Figures S12 and S13).

## 4. Conclusions

With the emergence of next-generation sequencing (NGS) technologies, whole-genome analysis in an unprecedented number of cells has become possible. As a result, these technologies generate large datasets characterized by ultra-high volume and complexity. To enable successful integration and overcome batch effects, several methodologies have been developed, drawing upon diverse concepts and approaches. These methods offer valuable solutions for harmonizing and effectively integrating multiple scRNA-seq datasets, facilitating comprehensive and accurate analyses across different experimental sources. Moving to the subsequent stages of feature selection, empirical evidence from prior research suggests that, amid the thousands of features in single-cell sequencing datasets, merely a few hundred predominantly influence the biological phenomenon under investigation. Our study aimed to underscore the efficacy and potential of refined feature selection strategies applied to single-cell data. Analyzing and interpreting big data, such as those derived from brain tissue, can provide valuable insights into the molecular and cellular processes underlying brain function and dysfunction, offering potential advancements in neuroscience and the understanding of neurological disorders.

Datasets were integrated through the use of Seurat’s CCA method and the provided lists were combined in a single consensus list through the use of the Borda rank-based count voting scheme. According to our analysis, we showed an involvement in glutamatergic synapses, dendrite and cell surfaces, regulation of synaptic vehicle exocytosis, regulation of norepinephrine secretion, and cerebral cortex GABAergic interneuron migration. In the hippocampus, DEGs were mainly enriched in the transcription of neuronal ligands, acetylcholine-related regulation of insulin secretion, neuropeptide hormones, and glutathione transferase activity. A comparison between the cortex and hippocampus revealed that DEGs were mainly enriched in muscarinic acetylcholine receptors, dopamine receptors, regulation of epinephrine secretion, and perisynaptic extracellular matrix. A comparison between the cortex and hippocampus of 6-month-old AD mice indicates that differential expressed genes (DEGs) were mainly enriched in central nervous system development and regulation of oligodendrocyte differentiation, whereas in 15-month-old AD mice, a significant enrichment was reported in regulation of epinephrine secretion and the regulation of dendritic spine morphogenesis. Data integration and feature selection between cortex and hippocampus tissues of young healthy mice revealed an enrichment in central nervous system development and regulation of oligodentrocyte differentiation. Further exploration and ML-based integration of scRNA-seq data across diverse brain regions could strengthen our pipeline and unravel additional molecular insights. Through single-cell transcriptomics, researchers have uncovered a wide array of molecularly diverse cell types within the nervous system. However, our comprehension of the lineage connections between mature cell types and progenitor cells remains limited. Addressing the challenges posed by the sheer size and complexity of data in genomics, DL and ML techniques can be efficiently implemented. These methods are well-suited for handling big data, leveraging their inherent capabilities to yield robust and reliable outcomes, especially in the context of large and intricate datasets generated by NGS technologies.

## Data Availability

Not applicable.

## Supplementary Materials

The following supporting information can be downloaded at: https://www.mdpi.com/article/10.3390/cimb45110544/s1, Figure S1. Gene annotation cluster networks. Visualizations generated for 100 top terms in related categories, with identified DEG lists and gene annotation cluster networks for GO cellular components; Figures S2–S4. Top 10 enriched BPs, CCs, and MFs for DEGs in 6-month-old cortex compared to 6-month-old hippocampus in control mice (datasets AD00301 and AD00702); Figures S5–S7. Top 10 enriched BPs, CCs, and MFs for DEGs in 6-month-old AD cortex compared to 7-month-old AD cortex (datasets AD00303 and AD00705); Figures S8–S10. Top 10 enriched BPs, CCs, and MFs for DEGs in 15-month-old AD cortex compared to 15-month-old AD hippocampus (datasets AD00307 and AD00714); Figures S11–S13. Top 10 enriched BPs, CCs, and MFs for DEGs in 15-month-old AD cortex compared to 15-month-old AD hippocampus (datasets AD00308 and AD00715); Table S1. Top 10 enriched Reactome pathways for DEGs in cortex; Table S2. Top 10 enriched biological processes for DEGs in cortex; Table S3. Top 10 enriched molecular functions for DEGs in cortex; Table S4. Top 10 enriched cellular components for DEGs in cortex; Table S5. Top 10 enriched Reactome pathways for DEGs in hippocampus; Table S6. Top 10 enriched biological processes for DEGs in hippocampus; Table S7. Top 10 enriched molecular functions for DEGs in hippocampus; Table S8. Top 10 enriched cellular components for DEGs in hippocampus; Table S9. Top 10 enriched Reactome pathways for DEGs in cortex compared to hippocampus; Table S10. Top 10 enriched biological processes for DEGs in cortex compared to hippocampus; Table S11. Top 10 enriched molecular functions for DEGs in cortex compared to hippocampus; Table S12. Top 10 enriched cellular components for DEGs in cortex compared to hippocampus.
