# Supplementary material for: Machine Learning Analysis of Alzheimer’s Disease Single-Cell RNA-Sequencing Data across Cortex and Hippocampus Regions
Source: Curr Issues Mol Biol. 2023 Oct 28;45(11):8652–69. doi: 10.3390/cimb45110544 (PMC10670182; doi:10.3390/cimb45110544)

Supplementary Material

Machine Learning of Alzheimer's Disease single cell RNA-sequencing data across Cortex and Hippocampus Regions

Marios G. Krokidis*, Aristidis G. Vrahatis, Konstantinos Lazaros, Konstantina Skolariki, Themis P. Exarchos and Panagiotis Vlamos

*Correspondence: mkrokidis@ionio.gr

Table S1. The top 10 enriched Reactome pathways for DEGs in cortex

Index	Name	P-value	Adjusted p-value	Odds Ratio	Combined score
1	Abacavir Transmembrane Transport R-HSA-2161517	0.02475	0.3024	50.24	185.84
2	Synthesis Of GDP-mannose R-HSA-446205	0.02475	0.3024	50.24	185.84
3	G2 Phase R-HSA-68911	0.02475	0.3024	50.24	185.84
4	RUNX2 Regulates Chondrocyte Maturation R-HSA-8941284	0.02475	0.3024	50.24	185.84
5	Extrinsic Pathway Of Fibrin Clot Formation R-HSA-140834	0.02475	0.3024	50.24	185.84
6	Metabolism Of Amine-Derived Hormones R-HSA-209776	0.003594	0.2379	25.36	142.75
7	FLT3 Signaling Thru SRC Family Kinases R-HSA-9706374	0.02963	0.3024	40.19	141.43
8	PTK6 Regulates Cell Cycle R-HSA-8849470	0.02963	0.3024	40.19	141.43
9	GLI Proteins Bind Promoters Of Hh Responsive Genes To Promote Transcription R-HSA-5635851	0.03448	0.3024	33.49	112.77
10	Insulin-like Growth Factor-2 mRNA Binding Proteins (IGF2BPs/IMPs/VICKZs) Bind RNA R-HSA-428359	0.03448	0.3024	33.49	112.77

Table S2. The top 10 enriched biological processes for DEGs in cortex

Index	Name	P-value	Adjusted p-value	Odds Ratio	Combined score
1	L-alanine Transport (GO:0015808)	0.0005113	0.08230	81.20	615.40
2	Osteoblast Development (GO:0002076)	0.0005113	0.08230	81.20	615.40
3	Embryonic Eye Morphogenesis (GO:0048048)	0.0008709	0.08230	58.00	408.65
4	Mesoderm Development (GO:0007498)	0.0001109	0.07252	38.44	350.03
5	Glycine Transport (GO:0015816)	0.001085	0.08230	50.74	346.39
6	T Cell Chemotaxis (GO:0010818)	0.001085	0.08230	50.74	346.39
7	Response To Nitric Oxide (GO:0071731)	0.001322	0.08230	45.10	298.98
8	Regulation Of T Cell Chemotaxis (GO:0010819)	0.001862	0.1009	36.90	231.95
9	L-amino Acid Transport (GO:0015807)	0.0002900	0.08230	26.73	217.71
10	Thiamine Transmembrane Transport (GO:0071934)	0.02475	0.2032	50.24	185.84

Table S3. The top 10 enriched molecular functions for DEGs in cortex

Index	Name	P-value	Adjusted p-value	Odds Ratio	Combined score
1	Glycine Transmembrane Transporter Activity (GO:0015187)	0.0008709	0.06663	58.00	408.65
2	Alanine Transmembrane Transporter Activity (GO:0022858)	0.002166	0.07319	33.82	207.50
3	Oxidoreductase Activity, Acting On Metal Ions, Oxygen As Acceptor (GO:0016724)	0.02475	0.1317	50.24	185.84
4	Thiamine Transmembrane Transporter Activity (GO:0015234)	0.02475	0.1317	50.24	185.84
5	ATP-activated Inward Rectifier Potassium Channel Activity (GO:0015272)	0.02475	0.1317	50.24	185.84
6	Amino Acid:Proton Symporter Activity (GO:0005280)	0.02475	0.1317	50.24	185.84
7	N,N-dimethylaniline Monooxygenase Activity (GO:0004499)	0.02475	0.1317	50.24	185.84
8	Acetylcholine Receptor Inhibitor Activity (GO:0030550)	0.02475	0.1317	50.24	185.84
9	Ferroxidase Activity (GO:0004322)	0.02475	0.1317	50.24	185.84
10	Basic Amino Acid Transmembrane Transporter Activity (GO:0015174)	0.003205	0.07319	27.05	155.37

Table S4. The top 10 enriched cellular components for DEGs in cortex

Index	Name	P-value	Adjusted p-value	Odds Ratio	Combined score
1	Smooth Endoplasmic Reticulum (GO:0005790)	0.004004	0.3604	23.87	131.77
2	HFE-transferrin Receptor Complex (GO:1990712)	0.03448	0.3954	33.49	112.77
3	Microfibril (GO:0001527)	0.05366	0.3954	20.09	58.77
4	Platelet Dense Granule Lumen (GO:0031089)	0.06310	0.3954	16.74	46.25
5	Cation-Transporting ATPase Complex (GO:0090533)	0.07246	0.3954	14.35	37.66
6	Chitosome (GO:0045009)	0.07246	0.3954	14.35	37.66
7	Melanosome Membrane (GO:0033162)	0.07246	0.3954	14.35	37.66
8	trans-Golgi Network Transport Vesicle (GO:0030140)	0.07246	0.3954	14.35	37.66
9	Pigment Granule Membrane (GO:0090741)	0.07246	0.3954	14.35	37.66
10	Protein Kinase Complex (GO:1902911)	0.08171	0.4086	12.55	31.44

Table S5. The top 10 enriched Reactome pathways for DEGs in hippocampus.

Index	Name	P-value	Adjusted p-value	Odds Ratio	Combined score
1	Synthesis Of Lipoxins (LX) R-HSA-2142700	0.02963	0.4184	40.19	141.43
2	Choline Catabolism R-HSA-6798163	0.02963	0.4184	40.19	141.43
3	Vitamin C (Ascorbate) Metabolism R-HSA-196836	0.03448	0.4184	33.49	112.77
4	MECP2 Regulates Transcription Of Neuronal Ligands R-HSA-9022702	0.03448	0.4184	33.49	112.77
5	Kinesins R-HSA-983189	0.001209	0.2550	15.75	105.81
6	Fatty Acids Bound To GPR40 (FFAR1) Regulate Insulin Secretion R-HSA-434316	0.03931	0.4184	28.71	92.90
7	Synthesis Of 5-Eicosatetraenoic Acids R-HSA-2142688	0.04412	0.4184	25.12	78.38
8	CD163 Mediating An Anti-Inflammatory Response R-HSA-9662834	0.04412	0.4184	25.12	78.38
9	Acetylcholine Regulates Insulin Secretion R-HSA-399997	0.04890	0.4184	22.32	67.37
10	Calcitonin-like Ligand Receptors R-HSA-419812	0.04890	0.4184	22.32	67.37

Table S6. The top 10 enriched biological processes for DEGs in hippocampus.

Index	Name	P-value	Adjusted p-value	Odds Ratio	Combined score
1	Kinetochore Organization (GO:0051383)	0.001085	0.2213	50.74	346.39
2	Negative Regulation Of Cation Channel Activity (GO:2001258)	0.0003628	0.1480	24.59	194.77
3	Regulation Of Epinephrine Secretion (GO:0014060)	0.02475	0.2707	50.24	185.84
4	ISG15-protein Conjugation (GO:0032020)	0.02475	0.2707	50.24	185.84
5	L-ascorbic Acid Metabolic Process (GO:0019852)	0.02475	0.2707	50.24	185.84
6	Glomerular Epithelial Cell Differentiation (GO:0072311)	0.02475	0.2707	50.24	185.84
7	Positive Regulation Of Mitotic Cytokinesis (GO:1903490)	0.02475	0.2707	50.24	185.84
8	Regulation Of Glucagon Secretion (GO:0070092)	0.02475	0.2707	50.24	185.84
9	Regulation Of Guanylate Cyclase Activity (GO:0031282)	0.02475	0.2707	50.24	185.84
10	Positive Regulation Of Receptor Binding (GO:1900122)	0.02475	0.2707	50.24	185.84

Table S7. The top 10 enriched molecular functions for DEGs in hippocampus.

Index	Name	P-value	Adjusted p-value	Odds Ratio	Combined score
1	Galactosidase Activity (GO:0015925)	0.02963	0.4260	40.19	141.43
2	Guanylate Cyclase Activator Activity (GO:0030250)	0.02963	0.4260	40.19	141.43
3	Tubulin-Glutamic Acid Ligase Activity (GO:0070740)	0.03931	0.4260	28.71	92.90
4	Neuropeptide Activity (GO:0160041)	0.007439	0.2350	16.90	82.83
5	Neuropeptide Hormone Activity (GO:0005184)	0.007439	0.2350	16.90	82.83
6	Glutathione Transferase Activity (GO:0004364)	0.008596	0.2350	15.60	74.20
7	Myosin II Binding (GO:0045159)	0.04890	0.4260	22.32	67.37
8	Phosphatidylinositol Phosphate 5-Phosphatase Activity (GO:0034595)	0.05366	0.4260	20.09	58.77
9	5'-Nucleotidase Activity (GO:0008253)	0.05839	0.4260	18.26	51.88
10	Aldehyde Dehydrogenase (NAD+) Activity (GO:0004029)	0.06779	0.4260	15.45	41.59

Table S8. The top 10 enriched cellular components for DEGs in hippocampus

Index	Name	P-value	Adjusted p-value	Odds Ratio	Combined score
1	Junctional Sarcoplasmic Reticulum Membrane (GO:0014701)	0.03931	0.3445	28.71	92.90
2	Condensed Chromosome, Centromeric Region (GO:0000779)	0.04412	0.3445	25.12	78.38
3	Axonal Growth Cone (GO:0044295)	0.07246	0.4150	14.35	37.66
4	Cortical Actin Cytoskeleton (GO:0030864)	0.02043	0.3445	9.65	37.54
5	Sarcoplasmic Reticulum (GO:0016529)	0.02131	0.3445	9.42	36.27
6	Kinetochore Microtubule (GO:0005828)	0.08631	0.4404	11.81	28.94
7	Tertiary Granule Lumen (GO:1904724)	0.02891	0.3445	7.94	28.15

Index	Name	P-value	Adjusted p-value	Odds Ratio	Combined score
8	AMPA Glutamate Receptor Complex (GO:0032281)	0.09088	0.4404	11.16	26.76
9	Mitochondrial Ribosome (GO:0005761)	0.1045	0.4537	9.56	21.60
10	Heterotrimeric G-protein Complex (GO:0005834)	0.1134	0.4537	8.73	19.00

Table S9. The top 10 enriched Reactome pathways for DEGs in cortex compared to hippocampus.

Index	Name	P-value	Adjusted p-value	Odds Ratio	Combined score
1	Signaling By Membrane-Tethered Fusions Of PDGFRA Or PDGFRB R-HSA-9673768	0.02475	0.2175	50.24	185.84
2	PTK6 Expression R-HSA-8849473	0.02475	0.2175	50.24	185.84
3	Tachykinin Receptors Bind Tachykinins R-HSA-380095	0.02475	0.2175	50.24	185.84
4	Muscarinic Acetylcholine Receptors R-HSA-390648	0.02475	0.2175	50.24	185.84
5	Dopamine Receptors R-HSA-390651	0.02475	0.2175	50.24	185.84
6	NGF-independant TRKA Activation R-HSA-187024	0.02475	0.2175	50.24	185.84
7	Synthesis Of Lipoxins (LX) R-HSA-2142700	0.02963	0.2175	40.19	141.43
8	PTK6 Promotes HIF1A Stabilization R-HSA-8857538	0.02963	0.2175	40.19	141.43
9	Activation Of TRKA Receptors R-HSA-187015	0.02963	0.2175	40.19	141.43
10	Defective B3GALT6 Causes EDSP2 And SEMDJL1 R-HSA-4420332	0.004434	0.1256	22.54	122.14

Table S10. The top 10 enriched biological processes for DEGs in cortex compared to hippocampus.

Index	Name	P-value	Adjusted p-value	Odds Ratio	Combined score
1	Negative Regulation Of Cardiocyte Differentiation (GO:1905208)	0.0003664	0.05163	101.51	803.12
2	Adenylate Cyclase-Activating Adrenergic Receptor Signaling Pathway (GO:0071880)	0.002491	0.1386	31.22	187.17
3	Positive Regulation Of Gap Junction Assembly (GO:1903598)	0.02475	0.2387	50.24	185.84
4	ERBB2-EGFR Signaling Pathway (GO:0038134)	0.02475	0.2387	50.24	185.84
5	Regulation Of Epinephrine Secretion (GO:0014060)	0.02475	0.2387	50.24	185.84
6	Regulation Of Establishment Of T Cell Polarity (GO:1903903)	0.02475	0.2387	50.24	185.84
7	Toll-Like Receptor 9 Signaling Pathway (GO:0034162)	0.02475	0.2387	50.24	185.84
8	Neurotransmitter Loading Into Synaptic Vesicle (GO:0098700)	0.02475	0.2387	50.24	185.84
9	Regulation Of Nitric Oxide Mediated Signal Transduction (GO:0010749)	0.02475	0.2387	50.24	185.84
10	Prepulse Inhibition (GO:0060134)	0.02475	0.2387	50.24	185.84

Table S11. The top 10 enriched molecular functions for DEGs in cortex compared to hippocampus.

Index	Name	P-value	Adjusted p-value	Odds Ratio	Combined score
1	1-Acylglycerophosphocholine O-acyltransferase Activity (GO:0047184)	0.02475	0.2301	50.24	185.84
2	Amino Acid:Proton Symporter Activity (GO:0005280)	0.02475	0.2301	50.24	185.84
3	Phospholipase D Activity (GO:0004630)	0.02475	0.2301	50.24	185.84
4	Coreceptor Activity Involved In Wnt Signaling Pathway, Planar Cell Polarity Pathway (GO:1904929)	0.02475	0.2301	50.24	185.84
5	Secondary Active Monocarboxylate Transmembrane Transporter Activity (GO:0015355)	0.02475	0.2301	50.24	185.84
6	G Protein-Coupled Acetylcholine Receptor Activity (GO:0016907)	0.02963	0.2301	40.19	141.43
7	G Protein-Coupled Neurotransmitter Receptor Activity (GO:0099528)	0.02963	0.2301	40.19	141.43
8	Nucleotide Transmembrane Transporter Activity (GO:0015215)	0.03448	0.2301	33.49	112.77
9	2-Acylglycerol-3-Phosphate O-acyltransferase Activity (GO:0047144)	0.03448	0.2301	33.49	112.77
10	Xylosyltransferase Activity (GO:0042285)	0.03448	0.2301	33.49	112.77

Table S12. The top 10 enriched cellular components for DEGs in cortex compared to hippocampus.

Index	Name	P-value	Adjusted p-value	Odds Ratio	Combined score
1	Perineuronal Net (GO:0072534)	0.0006796	0.02242	67.67	493.57
2	Perisynaptic Extracellular Matrix (GO:0098966)	0.0008709	0.02242	58.00	408.65
3	Multivesicular Body, Internal Vesicle (GO:0097487)	0.02475	0.1906	50.24	185.84
4	Neuronal Dense Core Vesicle (GO:0098992)	0.02475	0.1906	50.24	185.84
5	Ionotropic Glutamate Receptor Complex (GO:0008328)	0.0005934	0.02242	20.48	152.19
6	Chloride Channel Complex (GO:0034707)	0.02963	0.1906	40.19	141.43
7	AMPA Glutamate Receptor Complex (GO:0032281)	0.004004	0.08248	23.87	131.77
8	Clathrin-Sculpted Gamma-Aminobutyric Acid Transport Vesicle (GO:0061200)	0.03448	0.1906	33.49	112.77
9	Clathrin-Sculpted Gamma-Aminobutyric Acid Transport Vesicle Membrane (GO:0061202)	0.03448	0.1906	33.49	112.77
10	AP-2 Adaptor Complex (GO:0030122)	0.03931	0.1906	28.71	92.90

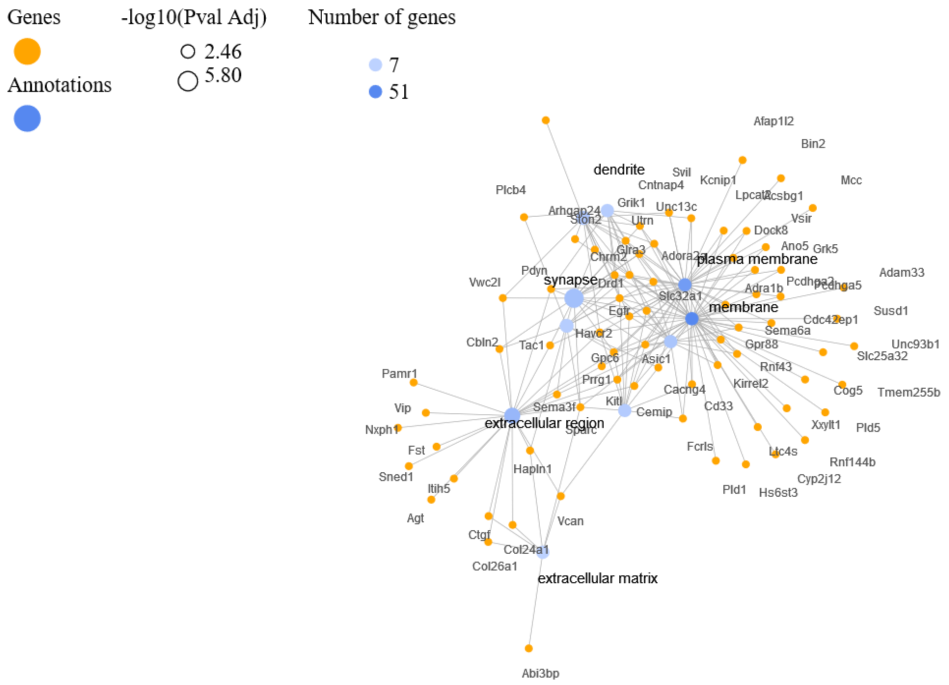

Figure S1. Gene-annotation cluster networks. Visualizations generated for 100 top terms in related categories with identified DEG list and gene-annotation cluster networks for GO cellular components.

Figure S2. Top 10 enriched biological processes for DEGs in 6-month-old cortex compared to 6-month-old hippocampus in control mice (datasets AD00301 and AD00702).

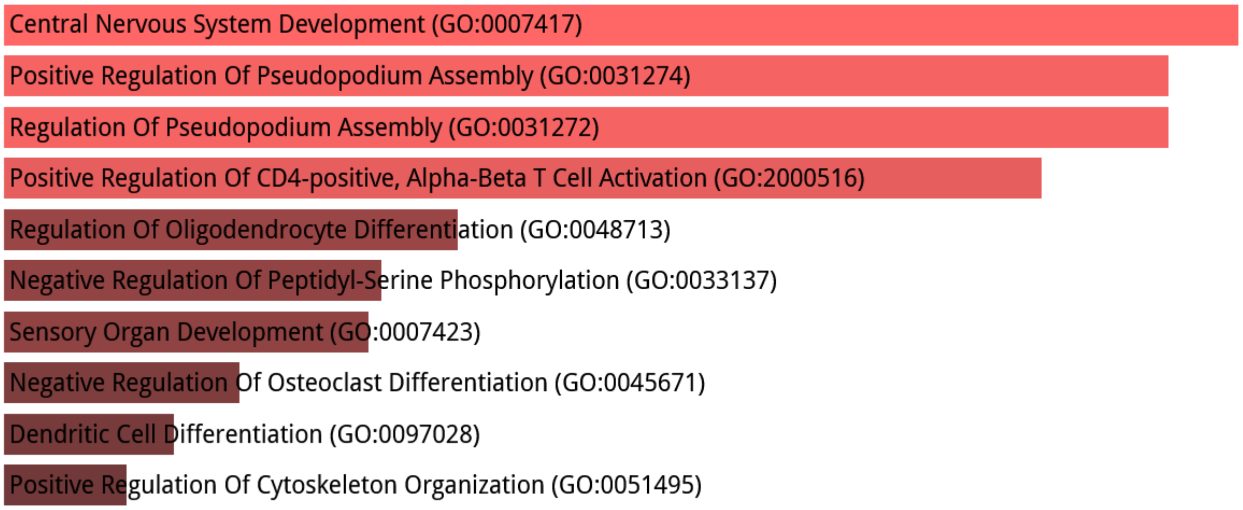

Figure S3. Top 10 enriched cellular components for DEGs in 6-month-old cortex compared to 6-month-old hippocampus in control mice (datasets AD00301 and AD00702).

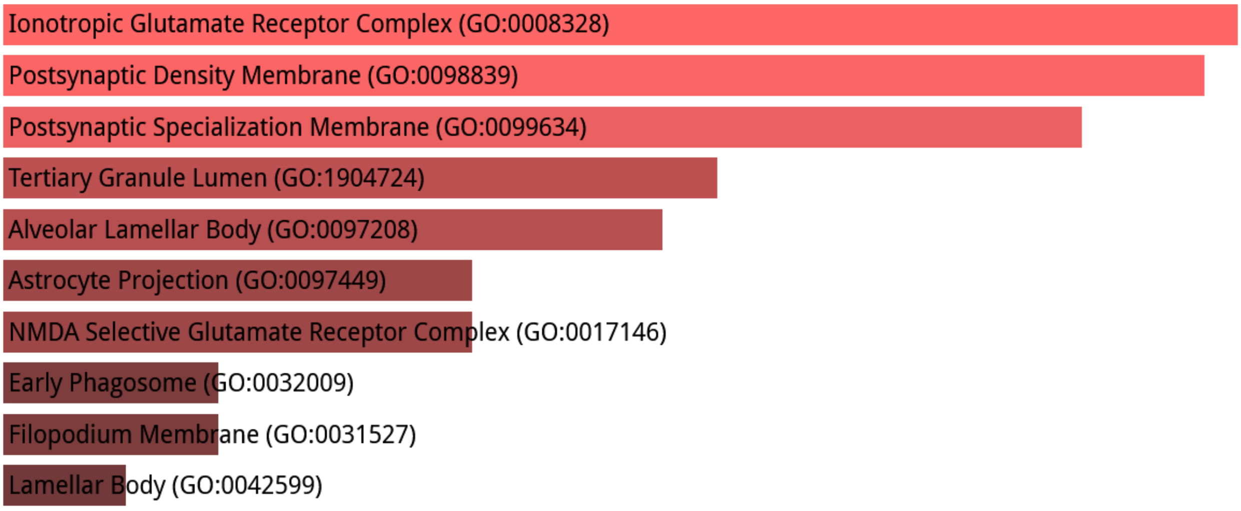

Figure S4. Top 10 enriched molecular functions for DEGs in 6-month-old cortex compared to 6-month-old hippocampus in control mice (datasets AD00301 and AD00702).

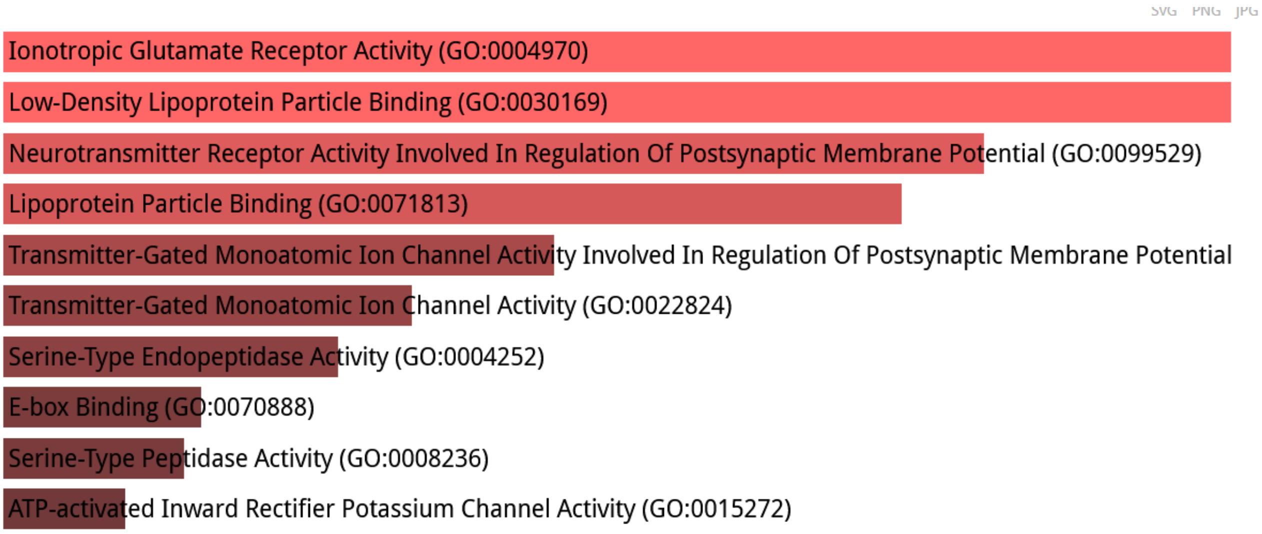

Figure S5. Top 10 enriched biological processes for DEGs in 6-month-old AD cortex compared to 7-month-old AD cortex (datasets AD00303 and AD00705).

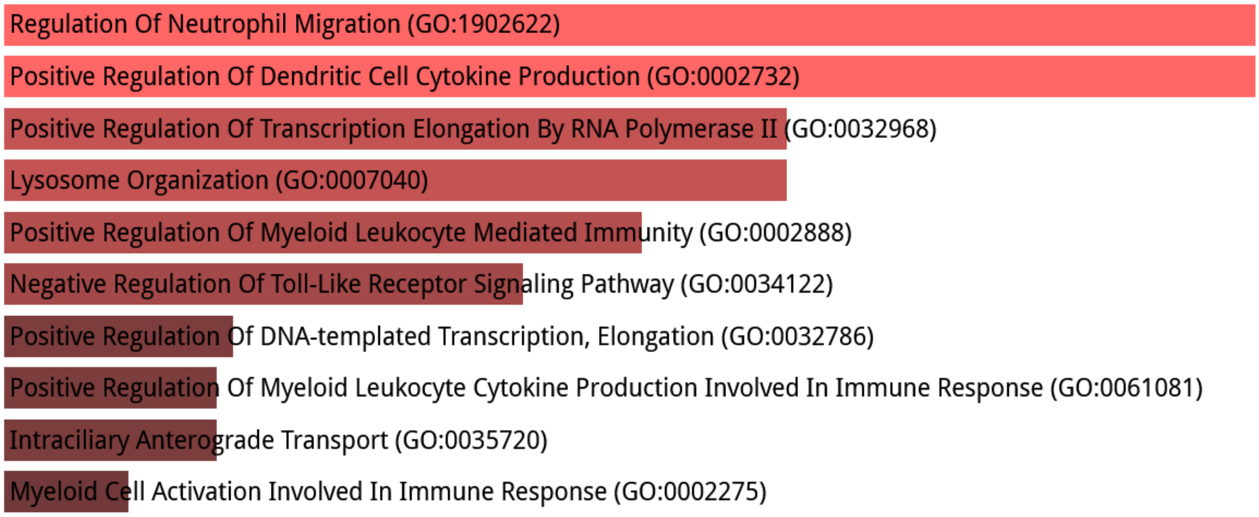

Figure S6. Top 10 enriched cellular components for DEGs in 6-month-old AD cortex compared to 7-month-old AD cortex (datasets AD00303 and AD00705).

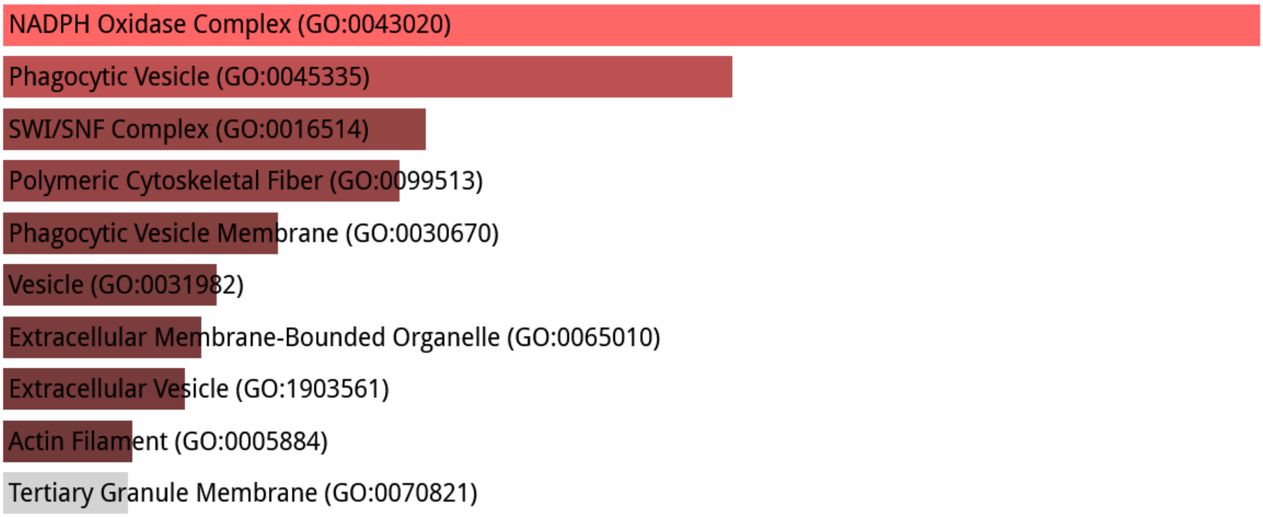

Figure S7. Top 10 enriched molecular functions for DEGs in 6-month-old AD cortex compared to 7-month-old AD cortex (datasets AD00303 and AD00705).

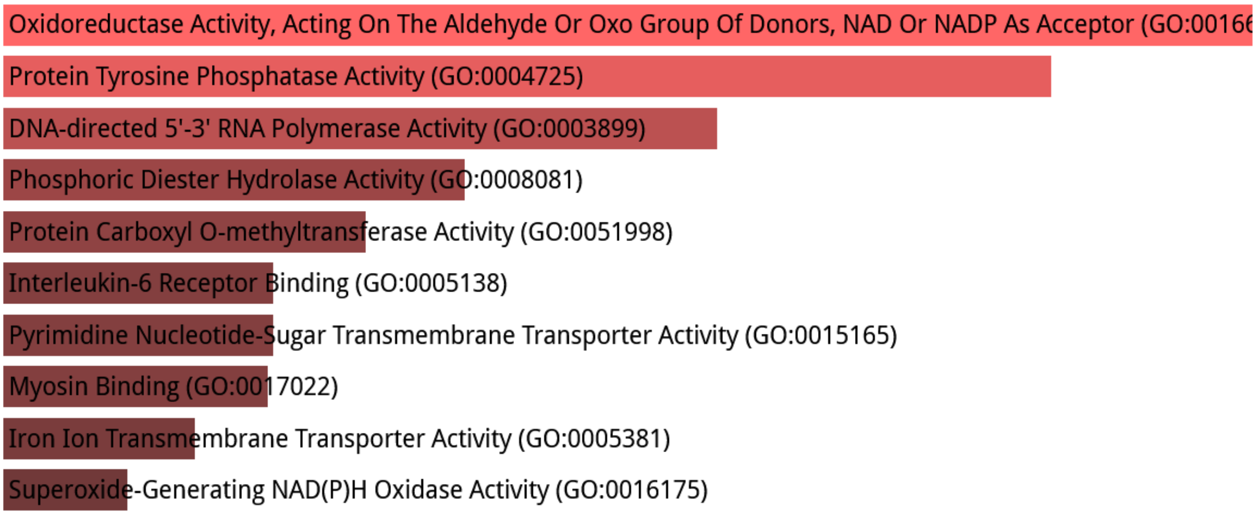

Figure S8. Top 10 enriched biological processes for DEGs in 15-month-old AD cortex compared to 15-month-old AD hippocampus (datasets AD00307 and AD00714).

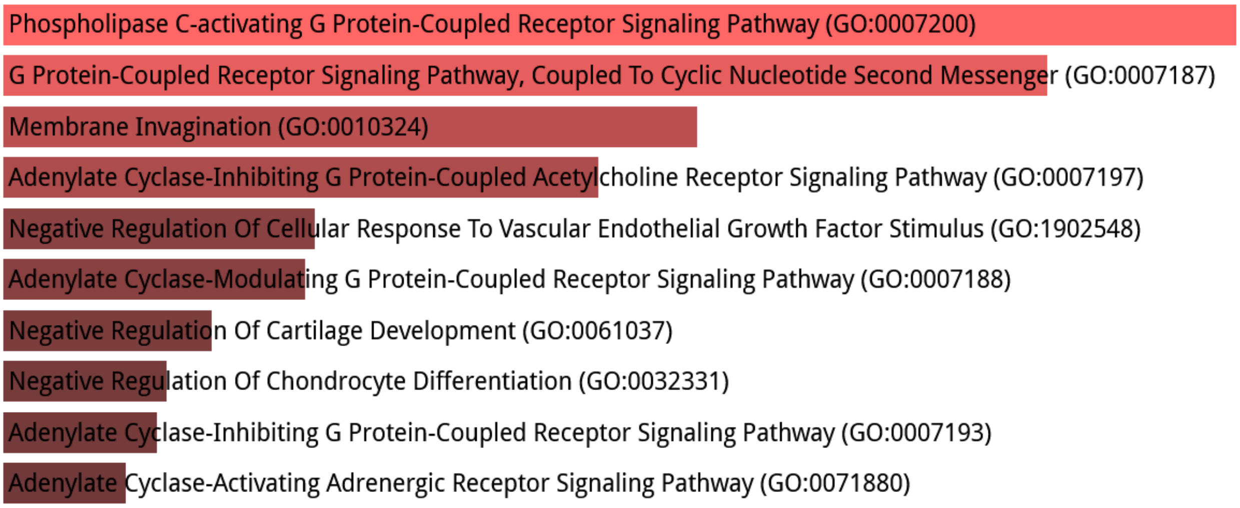

Figure S9. Top 10 enriched cellular components for DEGs in 15-month-old AD cortex compared to 15-month-old AD hippocampus (datasets AD00307 and AD00714).

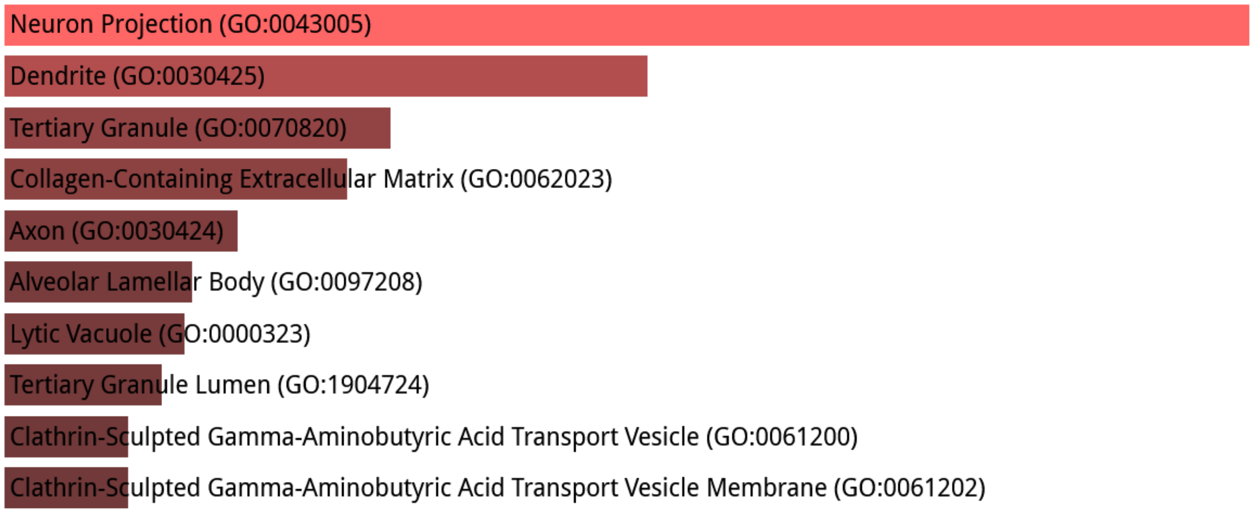

Figure S10. Top 10 enriched molecular functions for DEGs in 15-month-old AD cortex compared to 15-month-old AD hippocampus (datasets AD00307 and AD00714).

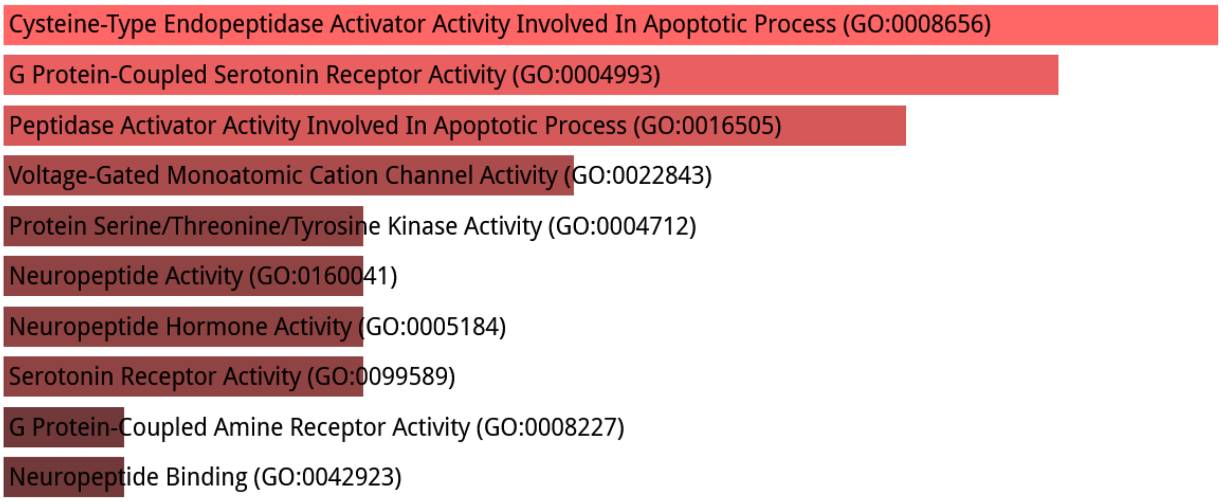

Figure S11. Top 10 enriched biological processes for DEGs in 15-month-old AD cortex compared to 15-month-old AD hippocampus (datasets AD00308 and AD00715).

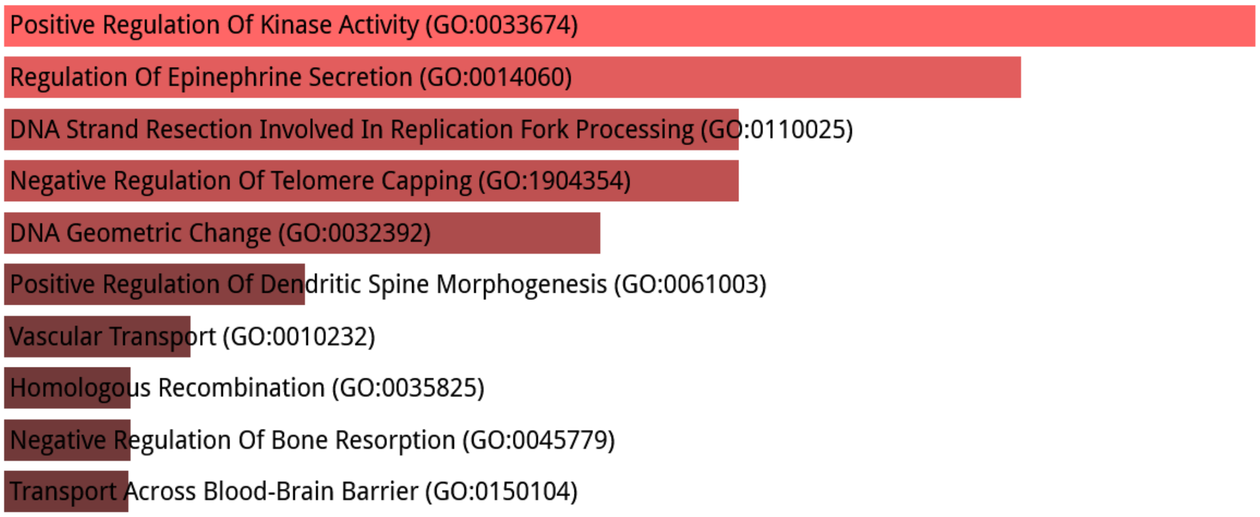

Figure S12. Top 10 enriched cellular components for DEGs in 15-month-old AD cortex compared to 15-month-old AD hippocampus (datasets AD00308 and AD00715).

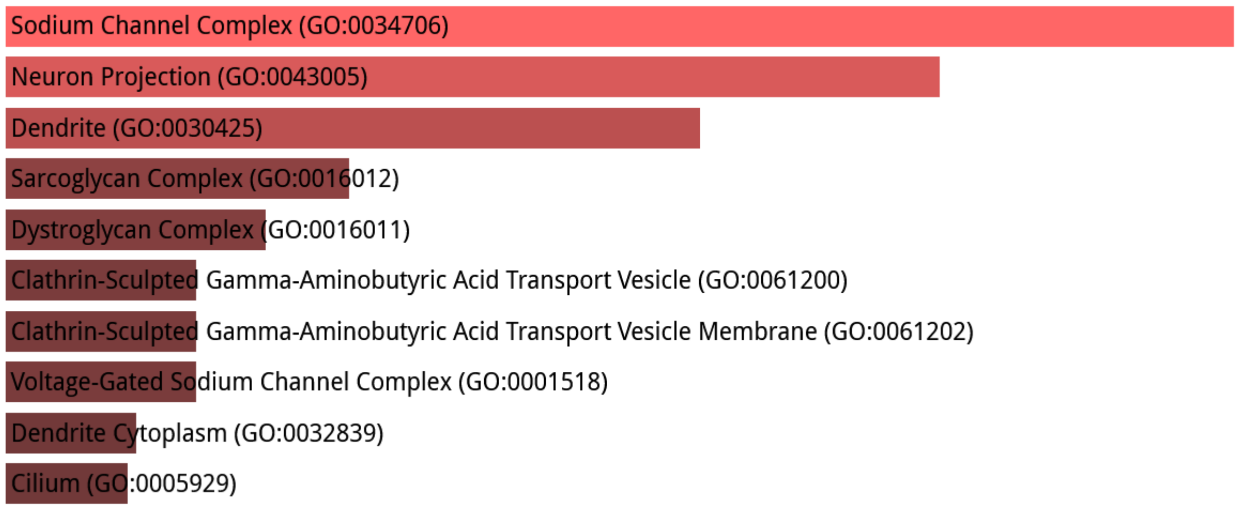

Figure S13. Top 10 enriched molecular functions for DEGs in 15-month-old AD cortex compared to 15-month-old AD hippocampus (datasets AD00308 and AD00715).

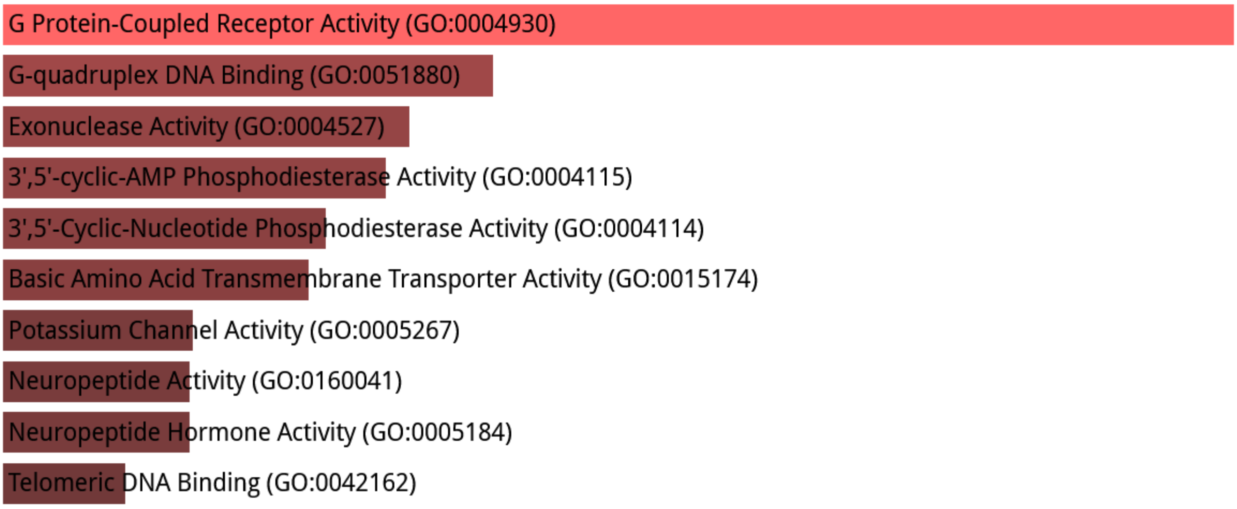

Supplement: Supplementary file 1 [file cimb-45-00544-s001.zip › cimb-2667946-supplementary.pdf]
